# Parents' experiences of diagnosis and specialist care of children's rare birthmarks

**DOI:** 10.1111/bjhp.70095

**Published:** 2026-07-15

**Authors:** Morgan B. Zolkwer, Satyamaanasa Polubothu, Veronica A. Kinsler, Amy Slater, Ella Guest

**Affiliations:** ^1^ Mosaicism and Precision Medicine Laboratory The Francis Crick Institute London UK; ^2^ Genetics and Genomic Medicine, UCL Great Ormond Street Institute of Child Health London UK; ^3^ Great Ormond Street Hospital Paediatric Dermatology London UK; ^4^ Centre for Appearance Research University of the West of England Bristol UK

**Keywords:** adjustment, arteriovenous malformation, congenital melanocytic naevi, dermatology, health care, rare birthmarks

## Abstract

**Objectives:**

Congenital melanocytic naevi (CMN) and arteriovenous malformations (AVM) are rare, severe and incurable birthmark conditions associated with lifelong visible difference and complex medical needs. Despite the importance of early health care experiences for parental and child adjustment in general, these remain unexplored in this context. This study aimed to explore parental experiences of raising a child with rare severe birthmarks, the role of specialist care in parental adjustment, and to develop recommendations for clinical care.

**Design:**

Reflective thematic analysis (RTA) was used to analyse semi‐structured narrative‐style interviews conducted with 23 parents of children aged ≤12 years recruited sequentially from a specialized NHSE Rare Disease Collaborative Network (RDCN) outpatient clinic in London, UK.

**Methods:**

Interviews were conducted virtually via telephone or Zoom and analysed using RTA using NVivo software (version 13). Reflexivity was achieved through the keeping of a reflexive journal and debriefing with the research team.

**Results:**

Through RTA, three themes were generated which highlight the challenges faced by parents of children with rare birthmarks. Firstly, parents' experiences before coming to the first appointment, secondly ‘learning to belong’ in the health care system and thirdly ‘making room’ for the condition. These themes and respective subthemes provide new insights into the importance of specialist centres in relation to parental adjustment to rare birthmarks.

**Conclusions:**

Findings underscore the pivotal role of specialist care in parental adjustment to rare, appearance‐altering conditions. Practical care recommendations are presented to support families across care pathways.

**Statement of Contribution**

**What Is Already Known on this Subject**

- Parents of children with rare conditions often struggle to achieve positive psychological adjustment.
- Parents of children with appearance‐altering conditions face additional psychological and social challenges when adjusting to parenthood when compared to parents of children without health conditions, or when compared to parents of children with rare conditions that do not alter appearance.
- Supporting positive parental adjustment to rare appearance‐altering conditions is essential for holistic care and benefits both parent and child wellbeing, yet this area has received little previous research attention.

**What Does this Study Add**

- To our knowledge, this is the first study to qualitatively explore parents' nuanced and complex experiences of the care pathway, diagnosis and specialist care in the United Kingdom for their child's rare birthmarks.
- Specialist care, particularly first specialist appointments, plays a pivotal role in shaping parental psychological adjustment to rare birthmarks.
- Optimized early specialist care presents a unique opportunity for health care professionals to facilitate long‐term positive parental adjustment, which in turn supports positive psychological adjustment of their child and family.

## INTRODUCTION

Having a child with a rare, chronic, appearance‐altering condition (defined as a condition that affects how someone looks; Rumsey & Harcourt, 2004) presents unique challenges beyond those faced by all parents (Jenkinson et al., 2015). Medically, these include attending hospital appointments and managing condition‐related uncertainty (von der Lippe et al., 2022). Socially, these include parental navigation of issues such as public curiosity, unsolicited comments or questions about the condition, and anxiety about the child's future social experiences and life engagement (Moss et al., 2020; Rumsey & Harcourt, 2004). Parental adjustment refers to overcoming these challenges effectively and is necessary to enable children to cope (Stock & Feragen, 2016). While some parents adjust well to having a child with a rare appearance‐altering condition, others find it challenging (Klein et al., 2006), and poor adjustment is linked to adverse psychological impacts including anxiety and depression in parents (Cohn et al., 2020). Two rare appearance‐altering conditions that have received little attention in the parental adjustment literature are congenital melanocytic naevi (CMN) and arteriovenous malformations (AVM), despite their severity and visibility. CMN and AVM are both examples of rare birthmark conditions. In the United Kingdom, both CMN and AVM are frequently managed within the Rare Disease Collaborative Network for Mosaic Disorders (RDCD‐MDs) specialist centres.

CMN and AVM are rare mosaic disorders, caused by sporadic in utero genetic mutations to the developing embryo or foetus (Al‐Olabi et al., 2018; Kinsler et al., 2013). Both are typically unsuspected before birth. CMN are moles on the skin which may also involve the central nervous system. This study focuses on parents of children with extensive or multiple CMN, which have an estimated prevalence of 1 in 10,000 newborns. CMN are visible at birth, may become much more numerous in childhood and are permanent. The skin can be lumpy, itchy and recurrently infected, and has an increased risk of melanoma development which requires monitoring. If melanoma develops in childhood, it is currently always fatal. Affected infants require MRI of the brain and spine in the first few months of life to check for neurological involvement, and if present children may develop seizures, hydrocephalus and developmental delay. AVM are high‐flow vascular malformations where arteries directly connect to veins and bypass the capillary bed. AVM are not always visible at birth, becoming noticeable over the first few years and continuing to progress thereafter. Complications include spontaneous high‐pressure bleeding with risk of sudden death, stroke, pain, loss of vision or other functions depending on the site and heart failure (Al‐Olabi et al., 2018). There are little data regarding the exact prevalence of AVM but they are known to be extremely rare (Liu et al., 2010).

Parents of children born with appearance‐altering conditions often experience initial shock, distress, isolation and guilt regarding the cause of their child's condition (Stock & Rumsey, 2015; Thornton et al., 2021), and that stress and uncertainty are heightened during the diagnostic phase (Gunter & Duke, 2018), and then again when children start to take responsibility for their condition management during adolescents (Day et al., 2025). A review of nine qualitative studies related to parental experiences of eczema, psoriasis and epidermolysis bullosa highlighted that stress related to physical care of skin conditions was frequently reported by parents (Ablett & Thompson, 2016). Parents of children with rare conditions face additional distress from underdeveloped care pathways, limited medical understanding and scarcity of treatments, resulting in inconsistent and challenging referral experiences (Tumiene & Graessner, 2021; von der Lippe et al., 2022). The recommended care pathway for children born with CMN or AVM in the United Kingdom and Europe is to be referred to national specialist multi‐disciplinary teams (MDTs) by paediatricians or general practitioners, upon the first presentation of the condition, usually at birth (Kinsler et al., 2020; NHS, 2023), however in practice the journey to specialist services is often very slow due to misdiagnosis. Specialist MDTs monitor CMN and AVM; symptomatic treatments and surgical procedures are often required, but there is no cure for CMN or AVM. Patients with CMN and AVM are initially seen by RDCN‐MDs clinicians on referral, with follow‐up scheduling depending on symptoms and prognosis. A typical patient follow‐up schedule for CMN or AVM is twice a year visit to the specialist centre in the first year followed by annual visits, with similar numbers of visits to the local hospital, however there can be many more appointments depending on the case.

High health care satisfaction has been demonstrated to protect against poor parental adjustment (Feragen et al., 2020; Thornton et al., 2021), and positive interactions with health care professionals (HCPs) aid parents in regaining control and accepting their child's condition (Kratz et al., 2009; Nelson et al., 2012). In particular, early experiences with HCPs shape future relationships in health care settings (Boshoff et al., 2018), suggesting that the initial specialist appointment may be important for parental adjustment. Despite this, little research has examined parents' experiences of their child's diagnosis and first specialist appointment for rare appearance‐altering conditions. We sought to gain novel insights from this patient cohort, with the aim of optimizing the care pathway to support both parental and child adjustment. CMN and AVM were investigated together because both are rare, incurable birthmark conditions associated with appearance‐altering features and severe, often non‐visible symptoms. Both are managed by the same MDTs within RDCN‐MDs specialist services, can be life‐threatening in severe cases, and remain underrepresented in the literature. Consultation with patient and public involvement (PPI) stakeholders, including CMN and AVM charities, supported investigating the conditions together due to their overlapping clinical, service‐related and psychological challenges. Psychological research also indicates that different appearance‐altering conditions are experienced similarly, and this has led the field to develop cross‐condition interventions for parents of children with appearance‐altering conditions (Rumsey & Harcourt 2004; Thornton et al., 2026).

## METHODS

### Design

An inductive qualitative design with semi‐structured, narrative‐style interviews was used to explore diagnostic experiences of having a child with CMN or AVM and wider parental adjustment. This study was underpinned by critical pragmatism, whereby qualitative methods were used to generate findings that were both theoretically informed and practically meaningful for families affected by rare birthmarks.

### Procedure

A semi‐structured narrative‐style interview schedule was developed based on existing literature on the psychological impact of parenting a child with a visible difference.

A patient and public involvement group consisting of members of relevant patient support groups reviewed the interview schedule and suggested minor adjustments. Further refinements were made based on feedback from two consultant paediatric dermatologists and a research fellow in appearance research, integrating lived experiences, clinical perspectives and theoretical insights. See Table 1 for the final interview schedule.

**TABLE 1:** Finalized interview schedule.

Please could you tell me about when you found out you that your child had an appearance‐altering condition?
2 Could you tell me about your journey from initially finding out your child had an appearance‐altering condition to your first specialist dermatologist appointment?
3 When you came to your initial specialist dermatologist appointment, what were you most hoping to come out of the appointment knowing?
4 Please could you take me through what you remember about your first specialist appointment?
5 Was there anything that you think could have been included in the first appointment which would have made you feel more supported or more prepared for the future?
6 What was it like returning home after your first appointment?
7 How have things been since then to now?

### Recruitment and participants

NHS ethical approval was provided as part of the Great Ormond St Hospital Rare Dermatology Diseases Resource research project (22/LO/0848/AM01). All participants provided written informed consent prior to enrolment in the study.

Parents of children aged ≤ 12 years with CMN or AVM attending paediatric dermatology clinics at the RDCN‐Mosaic Disorders specialist paediatric centre were verbally invited to participate in this research when they attended their specialist appointment. They chose between an in‐person interview at the clinic or a virtual/phone interview at their convenience. Twenty‐three parents were interviewed. Their demographic information is recorded in Tables 2 and 3.

**TABLE 2:** Participant family roles.

	CMN	AVM	Total
Mother	9	8	17
Father	4	2	6
Total	13	10	23

**TABLE 3:** Participant ethnicity.

Ethnicity	Frequency
White	18
Asian	4
Mixed‐race	1
Total	23

*Note*: Participants were asked to self‐describe their ethnicity and gender. These were categorized using Census categories to ensure anonymity.

Exclusion criteria were parents whose children had CMN or AVM that were revertant or had been completely surgically removed.

Interviews were carried out via Zoom Version 6.0 (Zoom, 2024; *n* = 3) or telephone (*n* = 16); four interviews involved both parents. Interviews ranged from 27 to 66 min (M = 43 min).

### Analysis

Interviews were recorded, transcribed verbatim, anonymised and analysed in NVivo Version 13 by the lead author using reflexive thematic analysis (RTA) to identify common experiences within the data (Braun & Clarke, 2006; Lumivero, 2020).

Reflexivity was achieved by means of keeping a reflexive journal during the whole study process, including after interviews and analysis, and debriefing with the research team. This included the authors reflecting on their positionalities and professional and personal experiences positionality statements are included in supportive information, and through practicing reflexivity within the research group regarding how identities and past experiences were shaping the analysis (Braun & Clarke, 2006). The research team acted as a ‘critical friend’ during this time by encouraging deeper reflections on how the researchers' perspectives shaped the analysis, especially after initial themes were generated, and then throughout the further analysis by asking probing questions about analytic decisions.

## FINDINGS

Three themes were generated: (1) Facing the unknown: Parents' uncertainty before the first appointment, (2) learning to belong in the health care system, (3) making room for CMN or AVM: adapting, connecting and coping. Respective subthemes were generated through RTA relating to the experiences of the parents.

### Theme 1: Facing the unknown: Parents' uncertainty before the first appointment

Theme 1 explores the emotions and pre‐conceived ideas that parents of children with CMN and AVM bring to their first specialist appointment and has three subthemes: ‘1.1: The isolation of being a parent of a child with a rare condition’, ‘1.2: The specialist centre as a unique place for a unique population of children’, ‘1.3: The overwhelming nature of being seen for the first time at a specialist centre’.

### Subtheme 1.1: The isolation of being a parent of a child with a rare disease

Some parents found the birth of their child distressing as this was when they first noticed their child's CMN or AVM, as discussed by Participants 3 and 18:

The doctors in the [birthing] room [at the local hospital] didn't say anything so [the father] was just really shocked so he thought like half of her body isn't working P3, mother, CMN.

The birthing team often had a lack of knowledge of CMN, due to its rarity, which contributed to feelings of shock and isolation and prompted self‐led research:

I came home and googled [the symptoms], I didn't get a good response from the website because it was so scary and I was reading some peoples' experiences, so I just closed my mobile and left it P18, mother, AVM.

Most parents had not previously contemplated their child looking different, as recounted by Participant 8:

It's shocking because when you're pregnant you go through all sorts of things that could be wrong but never thought of any sort of…physical differences P8, father, CMN.

For AVM, which is not always apparent at birth, distress emerged when parents noticed concerns in their child's early years. Parents reflected that isolation grew when they experienced unsatisfactory referral pathway experiences, such as not feeling listened to or having their concerns escalated for prolonged periods of time:

I kept taking her back [to the GP] because I wasn't satisfied [with the misdiagnosed AVM as a skin infection] and then eventually I changed doctors' surgery …the frustration at not being listened to for the first four/five years, that was huge, that was awful P21, mother, AVM.

After noticing their child's condition, some parents socially withdrew due to society's lack of knowledge about rare conditions and the resulting stigmatizing behaviour:

It's been really traumatic because I feel like I can't even take her out without people staring or asking questions P3, mother, CMN.

The description of unwanted curiosity causing trauma highlights this parent's desire for privacy and a sense of normality as they navigated the initial emotions of having a child who looked different. It underscores how the visibility of the condition can turn routine activities into sources of distress rather than comfort.

### Subtheme 1.2: The specialist centre as a unique place for a unique population of children

Before the first specialist appointment, most parents perceived the specialist centre as a hospital for severe illness. Due to previous misdiagnoses or mild initial symptoms, some parents felt their child was not ‘sick enough’ for specialist care leading to hesitation in participating in appointments due to guilt and the belief that specialist time should be reserved for sicker children, as indicated by Participant 22:

I know it sounds really silly as a parent but I used to kind of feel like she didn't warrant to be there … there would be so many sick children with cancer and no hair and my daughter is stood there kind of you know playing with the toys and I think I just felt a little bit bad so I probably held back and didn't want to go like an over sensitive parent when there are other parents there with dying children P22, mother, AVM.

The parents that perceived their child as not fitting the mould of who should be treated at the specialist centre experienced a disconnect between their perception of their child and the reality that their child had a rare condition. For parents who experienced an alarming first encounter with the condition, like noticing extensive CMN at birth, arriving at the specialist centre brought hope, as these parents hoped specialists could resolve these feelings:

From our local hospital we were told ‘they are the specialists they'll be able to help you’ so that's what we were just hopeful for, like between her birth and that [first specialist appointment] we were just holding onto the idea that maybe there is [a cure] out there P3, mother, CMN.

### Subtheme 1.3: The overwhelming nature of being seen for the first time at a specialist centre

Most parents felt overwhelmed when trying to accept their child's condition needed specialist care, fuelling anxiety and stress when coming to the specialist hospital for the first time:

We went to the specialist centre because obviously dealing with this first hand and like never ever seeing [a CMN before] we don't know what an earth is going on like what to expect or anything P3, mother, CMN.

Having endured much uncertainty throughout the referral pathway, anxiety was heightened by the desire for diagnosis and treatment related information so that they can start to picture what their future will look like:

I think [I was] probably just [hoping for the] sort of the obvious ones like if this doesn't fade or go how is she, how do you help her cope with having such a visible difference CMN P13, mother, CMN.

The logistical burden of visiting the specialist centre added to stress, perhaps due to additional environmental uncertainty which underscored their already uncertain states:

We're from [very far away] so it was a long way to go to hear bad news, to come all the way so we were really anxious P14, father, AVM.

Despite this, some parents recalled masking their anxiety to support their child's trip:

My daughter was fine she loved it she had the best time; she told all her friends, it was like awesome for her she was not stressed out at all … we kind of try to make a day trip of it when we go…we [as parents] were like proper trying to just hide anxiety P22, mother, AVM.

The differing emotions when coming to the first appointment between Participant 22 and their child highlights their contrasting attitudes towards the birthmark: The child understood it as part of themselves, while the parent did not understand and was anxious for their child.

Parents often experienced initial and sustained feelings of isolation. Positive referral experiences helped restore hope through strong bonds with HCPs. Conversely, negative experiences left parents to independently research symptoms, increasing distress. The specialist centre's reputation and parents' emotional responses to their child's condition influenced their initial sense of belonging at the RDCN hospital, leading to emotionally charged first specialist appointments.

### Theme 2: Learning to belong in the health care system

Theme 2 relates to the parents' experiences of being with a specialist for the first time and receiving appropriate care. Theme 2 has 3 subthemes: ‘2.1: Living appointment to appointment’, ‘2.2: People who know how to care’, ‘2.3: The desire to take the expertise home’.

### Subtheme 2.1: Living appointment to appointment

Before their child was seen in the specialist centre, parents felt like health care nomads, enduring chronic stress from inconsistent referral pathway experiences, as indicated by Participant 4:

It feels like you've got this rope around your neck and it's on then it's off, is it today no its not today … you can never really relax and even when you survive the appointment you've then got the results … P4, mother, CMN.

The simile ‘rope around your neck’ reflects the constant anxiety and uncertainty parents experience, showing how they are unable to fully relax between appointments.

I think that's kind of the trauma I think of living with this every day and it's that low level stress that's always there P4, mother, CMN.

Some parents took a few appointments to feel settled, as indicated by Participant 5 who discussed the ongoing development of the parent‐HCP alliance at the specialist centre:

We went for this appointment where blood tests were taken … I saw that a load of his blood tests were off and I thought hmm [the blood test results are] quite off compared to previous ones but no one's called me so I'm assuming they're not that worried… P5, mother, CMN.

This reflects parents' active engagement with medical information and reliance on reassurance from HCPs to manage their uncertainty.

…but then these appointments come and then the results come and then often it's like meeting [a new member of the team] … so again it's trying to build up that therapeutic relationship P5, mother, CMN.

This highlights the ongoing process of building trust with HCPs and the value of continuity in supporting therapeutic relationships. Other parents felt secure in their therapeutic relationship immediately, reflecting the range of experiences at this stage in parental adjustment, as indicated by Participant 17:

I feel like they will fix her and we've only had that one appointment and they have my full trust P17, mother, AVM.

Despite receiving concerning news at the first specialist appointment, such as potential central nervous system involvement for CMN, or the progressive nature of AVM, most parents were initially primarily focused on understanding and combatting the condition:

scary [returning home] but … I felt ready for the events coming I didn't feel at all insecure or worried, we were on the train ready to fight it yeah P23, mother, AVM.

When at the specialist centre for the first time, most parents still saw the conditions as an external adversary and valued the initial encounter with specialists as a crucial step in preparing for their anticipated battle.

We just kind of came out of there like totally amazed and really positive and really upbeat like they are going to fix my child, and she is going to go onto have a normal life without so much pain P17, father, AVM.

Other parents acknowledge how this first specialist appointment served as a ‘turning point’ (P13, mother, CMN) towards acceptance of their child's condition:

I think I was starting to think that I wouldn't get [the birthmark] removed but I was still kind of wanting to get it removed for just cosmetic reasons so maybe it was the start of me not wanting it removed because of that conversation P12, mother, CMN.

This may reflect how parents often initially struggle to integrate their child's condition into their overall view of their child. Over time, however, HCPs can support parents to recognize that their child and their child's condition are one. This helps parents to allow their feelings of love and connection to flow through their child's rare birthmark, rather than around it, fostering a more integrated understanding of their child's identity.

### Subtheme 2.2: People who know how to care

Meeting HCPs who had seen these conditions before made parents feel relieved and less isolated:

More of a relief to be honest because it was worrying when you hear you know they haven't seen it before [during the referral pathway] … but then as [the specialists explained] what it was and kind of explained you know what can happen in terms of it more than likely not going to affect her life it was a more reassuring deal P9, mother, CMN.

Being with HCPs who took accountability for their child's needs provided reassurance and a sense of being in the right system. This allowed parents to share their advocacy role and begin their journey of positive adjustment:

To be honest, someone just actually committing to knowing what it was was so nice because we'd had nothing for so many years so I think it was more kind of sad that she had something but more relief that it was manageable and someone actually knew rather than us walking away thinking oh gosh still we don't know what it is P18, mother, AVM.

P18's discussion about the relief they felt with the specialists pointed to the stark contrast between the anxiety experienced during the referral pathway and the relief felt during the first specialist appointment. This was echoed by families requiring multi‐disciplinary team (MDT) care:

We were having to carry all these around with us and everything going this person said this, whereas at [the RDCN centre] was just one place and everyone sort of came to us which was just really good P22, mother, AVM.

Some parents felt specialist appointments were overly routine and conflicted with desire for more time with the specialists, their need for reassurance and acknowledgment that their distress was justified, as indicated by Participant 3. Other parents found the routine and reassuring nature calming:

…for the doctors its nothing new to them because they've seen many children with these like this condition so it doesn't kind of phase them in a way but obviously as a parent whose child has got this and has no information about it it's just like you should inform the parent more and kind of go through it in more detail’ P3, mother, CMN.

‘They were just very casual and calm about it and reassuring P12, mother, CMN.

These different experiences indicate the complexity of emotions that parents bring to the first appointment. They illustrate how some parents want their need for information to be matched and validated by HCPs, perhaps as a way of offloading their difficult feelings, whereas others prefer their heightened state to be lowered by HCP's calmness and reassurance.

### Subtheme 2.3: The desire to take the expertise home

Most parents found the first appointment both informative and challenging. Parents valued receiving written information to help with the rapid pace and depth of the discussion:

I wouldn't have minded getting it all in writing like I did just nod my head a bit as he went through the stages of the different options and next steps and what to do but I did feel like it was clear what the plans were and what would be done P14, father, AVM.

Most parents desired a key contact to help consolidate knowledge and answer new questions, reducing reliance on their memory of the emotional and technical details:

I think to have a person you know you can communicate with I think would go a huge way to alleviating the anxiety of families P5, mother, CMN.

Parents believed a key contact could stop anxiety accruing between appointments, highlighting the positive psychological impact of appointments and the challenge of maintaining this remotely. A key contact could offer a place to share difficult emotions, reducing the burden parents carry alone.

The first specialist appointment was a unique experience for parents as it marked the end of the search for the right HCPs to care for their child. Parents often felt immense relief when meeting people who took responsibility for their child and who could answer their questions. This initial appointment helped parents begin their journey of positive adjustment. However, maintaining the psychological benefits of this first appointment was complex.

### Theme 3: Making room for CMN or AVM: Adapting, connecting and coping

Theme.

3 examines the new reality parents faced after the first specialist appointment and how they became better prepared to manage the impact of their children's condition. Theme 3 includes three subthemes: ‘3.1: Receiving care, finding support, reducing isolation’, ‘3.2: Fitting your life around the demands of the conditions’, ‘3.3: Other pieces of the puzzle’.

### Subtheme 3.1: Receiving care, finding support, reducing isolation

Parents felt less isolated and alone after meeting people who understood their child's condition, their experiences and who made them feel heard. However, negative social interactions: *‘In summer…they [strangers] look at you and asks what's wrong with your child's arm’* (P16, father, AVM), reminded parents of their separation from society:

[when people see my child's CMN] they haven't had time to process what's happened and they're going to feel really awkward and uncomfortable to ask, they're just going to go quiet and walk off and tell their friend “oh I've just seen a girl with spots all over her body but I don't know what it was” P2, father, CMN.

The knowledge gained in the first appointment helped parents cope and reduced the condition's psychological impact. Simplified explanations helped some parents manage curiosity but sustained society's lack of understanding:

I kind of keep it as simple as I can really because it is so complex … I just say it's a birthmark called an AVM and it's because there are tangled blood vessels that impact the blood flow P14, father, AVM.

Family and friends were a source of valuable support to most parents:

They're your family and I know close family are not going to give any kind of judgement…so I was quite comfortable talking to like my mum and my extended family P9, mother, CMN.

The emphasis on a lack of judgement suggests that parents often experience judgement in other contexts, making this a significant challenge at this stage of their journey. In this light, the non‐judgemental responses from family members can offer a crucial sense of sanctuary and relief. However, for others, family and friend's lack of understanding of the condition caused frustration:

I sort of had to justify [to the family] that we didn't have any genetic mutations in our family and that it's not because of anything, it's not a fault of ours P1, mother, CMN.

Support groups and social media influencers could provide a sense of community and condition‐related information:

I follow [an influencer with an AVM] on Instagram and I feel like I get a lot of background information from her videos, that is probably the only thing I would say that I have found helpful P21, mother, AVM.

Resources like Caring Matters Now's ‘How do you C Me Now’ exhibition and book fostered a positive and welcoming view of visible differences among parents, reducing perceptions of the condition as something to battle:

If you get proper resources [from support groups]…where other children have got it and you can see how parents are coping that kind of stuff is so important because it just lets you know you're not by yourself … and then your kids are not by themselves… they know that they're not like the odd one out and that they don't need to feel ashamed of having birth marks P9, mother, CMN.

Other parents avoided support groups due to potential exposure to negative information:

So we had different feelings about [Caring Matters Now], I was more of the I don't really want to get to involved because each case is a case and we don't have much information anyway, I just wanted to speak with specialists first…. P11, father, CMN.

This underscores how difficult it is for parents to come to terms with having a child with rare birthmarks, and how they can feel at their limits of negative emotion, and that anymore negative information would be too much.

My partner had a different view where she wanted to speak with everyone about their kids and their experiences P11, father, CMN.

However, for other parents, the extent of their difficulties means that they are searching for anything and everything to support them to cope. Having a child with a rarer phenotype constrained the benefits of support groups as parents were still effectively isolated from the group they belonged to on paper:

Particularly pertaining to appearance, I think because there were so many other things [requiring medical care] actually then I found it kind of irritating if I'm honest when I would join the Facebook groups [where some people were just concerned about the appearance altering aspect of CMN] P5, mother, CMN.

### Subtheme 3.2: Fitting your life around the demands of the conditions

Due to the varying phenotypes of CMN and AVM, some parents faced extensive practical challenges with their child's condition:

The difficulty [the AVM] causes is unreal you wouldn't believe it, you can't talk you can't eat you can't drink, every time it gets slightly knocked you're pouring out blood, yeah it was awful’ P15, mother, AVM.

‘I think what was hard about the colour and the appearance was when it started growing these nodules about a month later… kids with CMN have this [different genetic] causation and those tend to be the more nodular super itchy ones P5, mother, CMN.

Practical challenges of CMN or AVM could affect the whole family, causing parental guilt for restricting unaffected siblings' lives:

You know you want to take the kids on holiday you want to go and explore … but I feel at the minute our life is on hold we can't plan, we have to think is there a major hospital nearby P19, mother, AVM.

Some parents experienced anxiety about ensuring their child's needs were met when they couldn't directly oversee care:

I do worry about the sun cream because I'm easily burnt so that's the worry that it will get forgotten [at school] P10, mother, CMN.

This indicates how delicate a place it is when parents finally feel on top of their child's condition, and the dynamic nature of adjustment as their child progresses through life stages. Emotional challenges persisted for all parents after the first specialist appointment. Some were distressed by the severity and incurability of their child's condition. P3 (mother, CMN) felt *‘back to square one’* as initial hope clashed with the harsh reality of the diagnosis. Parents who were initially less concerned found the first appointment especially challenging upon learning the potential extent of the condition:

That's where we were told that if it's gone into his spine, he could have developmental delay…we were like ‘what how does this relate to our child?’…we had no reason to believe there was anything wrong with him when we went to that appointment P4, mother, CMN.

### Subtheme 3.3: Other pieces of the puzzle

Some parents noted that other life challenges helped them to re‐appraise their child's condition as less serious:

…but then my brother passed away not long after my child was born and it kind of put it into perspective P12, mother, CMN.

For other parents, existing stressors exacerbated initial negative emotions as parents felt overloaded:

Our dog at this point had lymphoma and was being treated for lymphoma so we had a lot on our hands at the time and when she was born it was more it's another problem we were having P11, father, CMN.

Some parents found local check‐ups reassuring and lessened their burden, while others experienced anxiety from conflicting opinions with specialists, complicating decisions.

I'm a bit reluctant going back [to the local hospital] as we did decide to go through [with the surgery that the local hospital didn't recommend] P13, mother, CMN.

Meeting others who understood their child's condition reduced parents' isolation, but negative interactions and rarer phenotypes reinforced separation. Nonetheless, with specialist care and increased time with their child, parents began positively adjusting. Understanding the condition helped most parents connect with their child and gain reassurance and hope from seeing their child develop alongside their peers. Gaining a care plan in the first appointment was especially valuable to all parents as they adjusted from feeling isolated and hopeless to feeling reassured and empowered.

## DISCUSSION

This qualitative study provides in‐depth insight into the experiences of parents of children with rare appearance‐altering conditions, CMN and AVM. The first specialist appointment appears to be pivotal in reassuring and empowering parents, helping them transition from chronic stress and uncertainty to security, hope and optimism. These findings emphasize the importance of this appointment in parental adjustment, aligning with literature on the diagnostic journey of other rare conditions (Bauskis et al., 2022; von der Lippe et al., 2022). The positive impact of confident, experienced specialists was evident, as the appointment helped parents integrate the condition into their lives and progress towards positive adjustment.

Initial negative emotions in parents of children with appearance‐altering conditions are exacerbated by the unfamiliarity of rare conditions and the inability to diagnose them prenatally (Stock & Rumsey, 2015; Thornton et al., 2021). The lack of awareness in the medical community sustained parent distress, highlighting the need to raise awareness of rare conditions medically and societally. This study underscores the importance of recognizing that families affected by rare conditions may have faced a challenging diagnostic journey before seeing specialists, and the negative effects of this need to be fully explored and considered by specialists (von der Lippe et al., 2022). Increased awareness could reduce parents' isolation and expedite specialist appointments, and positive interactions during the diagnostic journey might lessen the need for parents to advocate for their child (von der Lippe et al., 2022). The lack of public awareness about rare conditions made parents especially relieved by the first specialist appointment, which provided extensive information to counter negative social interactions and reduce social withdrawal (Germeni et al., 2018). This highlights the importance of organizations that increase awareness of rare conditions within health care settings.

In this study, highly stressed parents, often due to negative referral experiences, found the first specialist appointment most valuable. Conversely, some initially less concerned parents felt more distressed after the appointment. While previous research linked poor parental adjustment to depressive and anxious states and shows anxiety decreases post‐diagnosis (Cohn et al., 2020), this study reveals that more at‐risk parents may initially seem less distressed, which should be considered at the first appointment. This highlights the importance of checking and managing parents' expectations, especially at the first appointment. Parents who saw their child as ‘ordinary’ felt out of place at the specialist centre and struggled to accept the information provided. Acceptance, a core component of psychological interventions for caregivers of children with chronic appearance‐altering conditions, should be assessed and supported during consultations (Wright et al., 2023). At appointments, caregiver acceptance may be strengthened through the elicitation of strengths‐based narratives of the child, and where needed, referrals for interventions such as Tree of Life: a strengths‐based narrative therapy approach that uses the metaphor of a tree to help people reflect on their lives, identities and resilience (Haselhurst et al., 2021). Where these services are not available, other self‐help interventions based on Acceptance and Commitment Therapy, such as the Parenting Toolkit, have also demonstrated promise in promoting positive adjustment among parents of children with visible differences (Thornton et al., 2026). Parents initially viewed their child's condition as an external adversary, but with the optimistic support of specialists, they began to integrate the condition into their holistic view of their child. As they progressed through this process, their distress gradually subsided, highlighting the crucial role specialists play in fostering acceptance and promoting positive adjustment. Therefore, facilitating acceptance should be a key focus in specialist appointments. Future research should explore how preconceptions and prior experiences influence parents' care experiences and acceptance, with the goal of reducing distress, especially among parents who may appear initially outwardly resilient.

Most parents initially hoped for a cure, but for CMN and AVM, this was replaced by the realization of incurability. Specialists should communicate this challenging reality sensitively to promote a non‐pathological view, supporting parental adjustment, acceptance and assimilation of their child and the condition (López et al., 2021). Some parents with higher distress levels were more likely to seek appearance‐altering treatments, reflecting difficulties in adjusting and accepting its permanence (Thornton et al., 2021). This is supported by research on parental adjustment to childhood vitiligo, where parents described resigning themselves to the condition and therefore potentially more at peace (Moss et al., 2020). Reassurance during appointments sometimes felt overly routine; greater recognition of the condition's rarity and parental distress could strengthen parent–clinician relationships, as seen in paediatric oncology (Sisk et al., 2021). As discussed in this study and previous qualitative research, clinicians remain vigilant to the possibility of increased parental worry as children grow older and assume responsibility for condition management (Ablett & Thompson, 2016; Day et al., 2025). A contemporary finding related to post‐COVID hybrid health care is the increased reassurance provided by in‐person dermatology consultations. This preference likely arises from the visible nature of symptoms and parents' need to confirm that doctors have thoroughly observed them. This is supported by literature on patient satisfaction with other dermatological conditions (Lowe et al., 2022). Insights from the qualitative interviews and wider literature informed care recommendations for this population, which are summarized in the supportive information.

Theory and previous research suggest that parental adjustment to long‐term health conditions involves both positive and negative emotions, rather than simply the absence of distress (Thornton et al., 2021; Watson et al., 1999). Parents' accounts in this study support this view and highlight that adjustment is dynamic rather than linear, consistent with stage theories of adjustment (Wortman & Silver, 1989). Clinically, this emphasizes the importance of assessing and reassessing the interaction of positive and negative emotions when supporting parents at initial and subsequent appointments.

The findings should be interpreted in the context of several methodological considerations. The study, based on personal narratives, invited interpretive analysis due to its qualitative nature. This approach enhanced our understanding of the support needs of parents of children with CMN or AVM, the impact of current support on psychological adjustment, and potential improvements in care. Identification of relevant psychological constructs can inform quantitative research and merits further investigation in larger samples. The self‐selecting cohort did not overrepresent extreme experiences, as indicated by many unremarkable commonalities between different parents' experiences, and clinic‐based recruitment included under‐served populations.

## CONCLUSION

In‐depth explorations of parent experiences during the first specialist appointment for CMN or AVM have provided novel insights into parental experiences of rare birthmarks, the care pathway for these conditions in England, and the psychological, logistical and social burden associated with them. These findings also highlight key risk and protective factors influencing parental adjustment. Overall, this study underscores the crucial role of early, well‐coordinated specialist care in reducing distress, supporting acceptance and promoting positive long‐term adjustment for families affected by rare appearance‐altering conditions.

## AUTHOR CONTRIBUTIONS

**Morgan B. Zolkwer:** Conceptualization; investigation; writing – original draft; writing – review and editing; methodology; formal analysis. **Satyamaanasa Polubothu:** Writing – review and editing; data curation; supervision; resources. **Veronica A. Kinsler:** Conceptualization; investigation; funding acquisition; writing – review and editing; methodology; project administration; supervision; data curation; resources. **Amy Slater:** Investigation; conceptualization; writing – original draft; writing – review and editing; methodology; formal analysis; supervision. **Ella Guest:** Supervision; formal analysis; methodology; writing – review and editing; writing – original draft; conceptualization; investigation.

## Supporting information


**Data S1.** Supporting Information.

## Data Availability

The data that support the findings of this study are available from the corresponding author upon reasonable request.
