# Supplementary material for: Parents' experiences of diagnosis and specialist care of children's rare birthmarks
Source: Br J Health Psychol. 2026 Jul 15;31(3):e70095. doi: 10.1111/bjhp.70095 (PMC13373478; doi:10.1111/bjhp.70095)

**Supportive information**

1. **Author positionality statements**

The first author is a male in his mid-twenties and is not a parent. He has experience conducting interviews, but limited exposure to working with parents in a paediatric hospital setting. Through the interview process, the author deepened their understanding of the challenges faced by these parents. These insights informed and enriched this author's observations and reflections throughout the study. The second and third authors are both female dermatologists who work at Great Ormond Street Hospital. The fourth author is a woman in her late 40's with approximately 20 years of experience in appearance psychology. She is a parent and has some personal experience of having a critically ill newborn, although no experience of parenting a child with a rare birthmark condition. The fifth author is a woman in her early 30s. She is not a parent but has experience of carrying out qualitative research with parents of children with health conditions and has carried out qualitative research in the areas of health and appearance psychology for nine years.

1. **Care recommendations for medical staff supporting families affected by rare birthmarks to promote positive parental adjustment**


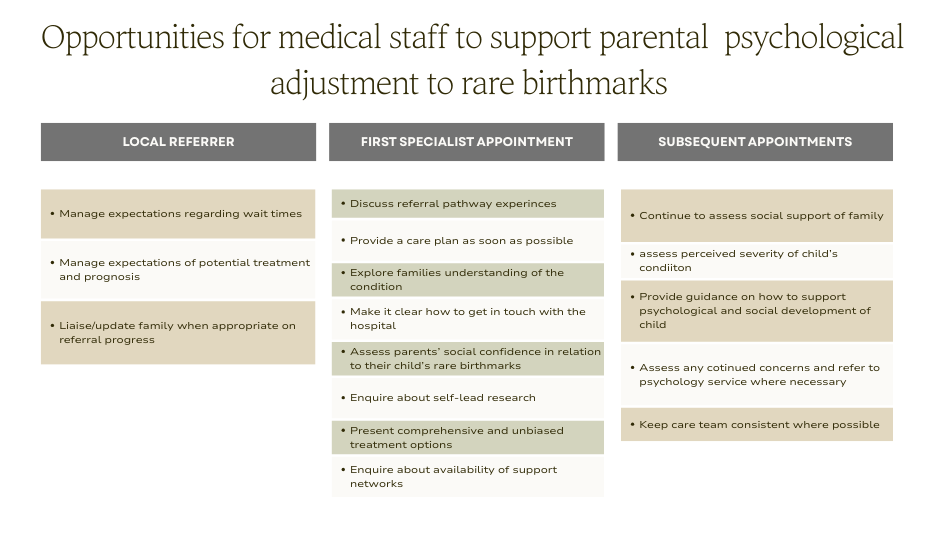

Supplement: Supplementary file 1 — Data S1. Supporting Information. [file BJHP-31-0-s001.docx]
